# Comparison of CAGE and RNA-seq transcriptome profiling using clonally amplified and single-molecule next-generation sequencing

**DOI:** 10.1101/gr.156232.113

**Published:** 2014-04

**Authors:** Hideya Kawaji, Marina Lizio, Masayoshi Itoh, Mutsumi Kanamori-Katayama, Ai Kaiho, Hiromi Nishiyori-Sueki, Jay W. Shin, Miki Kojima-Ishiyama, Mitsuoki Kawano, Mitsuyoshi Murata, Noriko Ninomiya-Fukuda, Sachi Ishikawa-Kato, Sayaka Nagao-Sato, Shohei Noma, Yoshihide Hayashizaki, Alistair R.R. Forrest, Piero Carninci, The FANTOM Consortium

**Affiliations:** 1 RIKEN Preventive Medicine and Diagnosis Innovation Program, Saitama 351-0198, Japan;; 2 RIKEN Omics Science Center, RIKEN Yokohama Institute, Yokohama, Kanagawa, 230-0045, Japan;^4^; 3 RIKEN Center for Life Science Technologies (CLST), Division of Genomic Technologies (DGT), Kanagawa, 230-0045, Japan

## Abstract

CAGE (cap analysis gene expression) and RNA-seq are two major technologies used to identify transcript abundances as well as structures. They measure expression by sequencing from either the 5′ end of capped molecules (CAGE) or tags randomly distributed along the length of a transcript (RNA-seq). Library protocols for clonally amplified (Illumina, SOLiD, 454 Life Sciences [Roche], Ion Torrent), second-generation sequencing platforms typically employ PCR preamplification prior to clonal amplification, while third-generation, single-molecule sequencers can sequence unamplified libraries. Although these transcriptome profiling platforms have been demonstrated to be individually reproducible, no systematic comparison has been carried out between them. Here we compare CAGE, using both second- and third-generation sequencers, and RNA-seq, using a second-generation sequencer based on a panel of RNA mixtures from two human cell lines to examine power in the discrimination of biological states, detection of differentially expressed genes, linearity of measurements, and quantification reproducibility. We found that the quantified levels of gene expression are largely comparable across platforms and conclude that CAGE and RNA-seq are complementary technologies that can be used to improve incomplete gene models. We also found systematic bias in the second- and third-generation platforms, which is likely due to steps such as linker ligation, cleavage by restriction enzymes, and PCR amplification. This study provides a perspective on the performance of these platforms, which will be a baseline in the design of further experiments to tackle complex transcriptomes uncovered in a wide range of cell types.

Measuring gene expression or transcript abundance is a key tool to study the regulation and molecular basis of biological systems. The emergence of next-generation sequencing technologies has enabled us to identify and quantify transcripts well beyond previous microarray-based technologies (Cloonan et al. 2008; Marioni et al. 2008; Mortazavi et al. 2008; ‘t Hoen et al. 2008; Suzuki et al. 2009; Valen et al. 2009; Levin et al. 2010; Plessy et al. 2010; Wu et al. 2010; Sam et al. 2011). The majority of transcriptome protocols running on second-generation sequencing platforms have relied on two PCR amplification steps: one for preamplication of cDNA and the other as clonal amplification of templates on the flow cell (or beads) prior to sequencing. These steps can generate potential bias in the identification and quantification of RNA molecules. With the use of single-molecule sequencers, it is possible to avoid PCR altogether (Kanamori-Katayama et al. 2011; Sam et al. 2011), thereby avoiding these potential biases.

RNA-seq is designed to identify transcript structure and abundance by sequencing randomly fragmented RNA or cDNA (Cloonan et al. 2008; Mortazavi et al. 2008). Several variations have been developed and have recently been compared (Marioni et al. 2008; ‘t Hoen et al. 2008; Sam et al. 2011). For second-generation sequencing using PCR, a ligation-based method is shown as the leading approach (Levin et al. 2010), whereas the HeliScope single-molecule sequencer reduced duplicated reads and avoided PCR biases altogether (Sam et al. 2011).

CAGE (cap analysis gene expression), on the other hand, was developed to identify and quantify 5′ ends of capped RNAs based on cap-trapping (Carninci et al. 1996). It originally employed Sanger sequencing (Shiraki et al. 2003; Kodzius et al. 2006) and was later adapted to 454 Life Sciences (Roche) and Illumina sequencers (Valen et al. 2009; The ENCODE Project Consortium 2011; Kurosawa et al. 2011; Takahashi et al. 2012). To increase the number of samples profiled while reducing the cost of sequencing, we developed barcoding strategies that allow the pooling of multiple libraries, combined sequencing, and later, separation based on the barcodes (Maeda et al. 2008; Kurosawa et al. 2011; Takahashi et al. 2012). Together, CAGE and RNA-seq have been employed in an extensive study of RNA subcellular localization by the ENCODE Project (Djebali et al. 2012), and the activities of transcription starting sites (TSSs) profiled by CAGE have also been used as a primary data source in the quantitative modeling of transcriptional output from epigenetic status (The ENCODE Project Consortium 2012). Recently, a simplified version of the CAGE protocol using a single-molecule sequencer, HeliScope, was developed; it avoids linker ligation, PCR, and enzymatic cleavage (Kanamori-Katayama et al. 2011). The HeliScope CAGE protocol (Kanamori-Katayama et al. 2011) has been used extensively to generate a promoter-level expression atlas across a diverse collection of mammalian cells in the FANTOM5 project (Forrest et al. 2014).

CAGE and RNA-seq identify different parts of RNA molecules: capped 5′ ends and random RNA fragments, respectively. Although dedicated experimental design is required to understand the performance of profiling technologies, the two approaches have not yet been systematically compared. Here we provide their systematic comparison, including variations of CAGE employing second- and third-generation sequencers (Illumina Genome Analyzer IIx and Helicos HeliScope) and RNA-seq on a second-generation sequencer. Based on a minimum unit of profiling, we examine technical reproducibility, expression level consistencies across platforms, and linearity of expression levels, as well as demonstrate their utility, with the aim of obtaining a less-biased perspective for practical use.

## Results and Discussion

### Experimental design and data production

**Figure 1.**
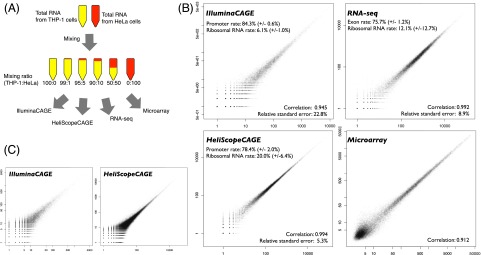
Experimental design and reproducibility. (*A*) Schematic representation of experimental design. (*B*) Scatter plots of quantified levels of gene expression and basic statistics. (±) Standard deviation. (*C*) Scatter plots of quantified levels of TSS activities at 1-bp resolution.

We applied three transcriptome profiling technologies based on next-generation sequencers—CAGE with a second-generation sequencer employing PCR preamplification prior to clonal amplification (IlluminaCAGE), CAGE with a third-generation, single-molecule sequencer skipping any PCR steps in all the steps (HeliScopeCAGE), and RNA-seq with Illumina GA IIx employing a ligation-based, strand-specific method—to the same series of RNA pools (Fig. 1A). For this we prepared total RNA extracted from THP-1 and HeLa cell lines and mixed them with different ratios (100:0, 99:1, 95:5, 90:10, 50:50, and 0:100 ratio of THP-1:HeLa). This asymmetric design could allow us to assess sensitivity in measuring differences between cellular expression profiles, even with a low amount of differentially expressed (<1%) “genes.” We profiled these RNA mixtures by each technology and microarray. One exception was RNA-seq, where only the four mixtures, 100:0, 99:1, 50:50, 0:100, were profiled, due to cost limitations, since a barcoding scheme was not available for this at the time. Although deeper sequencing (i.e., a larger amount of reads) is ideal for sequencing-based quantification methods theoretically, we performed each profiling within its minimum unit to understand what we can expect from the minimum profiling, that is, one channel or lane per one sample with HeliScopeCAGE and RNA-seq, and one lane per eight samples (multiplexing) with IlluminaCAGE. The number of replicates and obtained reads from individual profiles are summarized in Supplemental Table 1.

### Technical reproducibility

First we examined consistencies across technical replicates on the same platform. We counted reads mapping within 500 bases of an annotated 5′ end as expression from the transcript in CAGE, and reads mapping within exons of known transcripts as expression from the transcript in RNA-seq. Their fractions within the total reads indicate signal ratio, and the fraction of ribosomal RNA indicates unintended signals in both of the technologies. Figure 1B shows scatter plots of gene expressions between technical replicates obtained from THP-1 RNA with the fraction of the intended and unintended signals. IlluminaCAGE captured the highest signal fraction, 84% of the mapped reads coming from promoter regions, and variability of the promoter-hitting rate is comparably low in all platforms (standard deviation ≤2.0%) (Fig. 1B). The ability to exclude ribosomal RNA from other long RNAs is the highest in IlluminaCAGE, which is consistent with the results on the promoter ratio. The ribosomal RNA rate is the most variable in RNA-seq, which indicates that the selection of polyA-tailed RNAs by using oligo(dT) beads may be more variable than the selection of 5′ end capped RNAs.

The scatter plots in Figure 1, B and C, show that all of the platforms display high correlations between technical replicates at gene levels (Spearman’s correlation coefficient ≥0.9) and even at a single base pair of a TSS. Since the correlation coefficient itself is affected by sequencing depth (deeper sequencing data leads to higher correlation coefficients; in fact, the depth of IlluminaCAGE data is about one-tenth of HeliScopeCAGE and RNA-seq, as indicated in Supplemental Table 1), we estimated relative standard errors (standard deviation of expressions relative to average expression; square root of the estimated common overdispersion) by using edgeR (Robinson et al. 2010) as a metric of reproducibility performance independent of the sequencing depth. We found that estimated relative standard errors are low (≤10%) for RNA-seq and HeliScopeCAGE, whereas IlluminaCAGE profiles are relatively variable.

Taken together, this comparison suggests that the outputs of all the platforms are reasonably reproducible and that HeliScopeCAGE quantifies gene expression with the least variability, which could be explained by its simplified PCR-free protocol relying on a single-molecule sequencer (Kanamori-Katayama et al. 2011). Instead, IlluminaCAGE requires additional steps, including linker ligation, use of restriction enzyme, and PCR amplification. Optimization of individual steps likely contributes to enrich signal ratio but increases the variability in gene expression quantification. Since IlluminaCAGE employs the multiplexing strategy based on barcode sequences in linker oligonucleotides, one might expect bias across the barcodes in a similar manner to small RNA sequencing (Kawano et al. 2010; Alon et al. 2011). Scatter plots of gene expressions across replicates based on the same barcode sequence (Supplemental Fig. S1) demonstrate that variation across the barcodes is smaller than the one across operational preparations. This is consistent with a previous study, where barcode-based pooling contributes better reproducibility in CAGE employing the 454 Life Sciences (Roche) sequencer (Maeda et al. 2008). RNA-seq is highly reproducible at a similar level to HeliScopeCAGE; however, selection of polyA-tailed transcripts is relatively unstable. This could be improved by standardization of the selection procedure or by alternative approaches to deplete ribosomal RNA.

### Quantification of RNA mixtures

**Figure 2.**
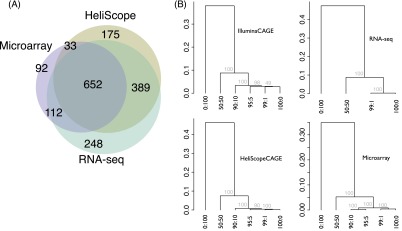
Gene expression quantification across different RNAs. (*A*) Venn diagram of up-regulated genes in HeLa cells against THP-1 cells. (*B*) Hierarchical clustering of the six RNAs, based on highly expressed 8000 gene expressions. Gray font indicates the reliability of the grouping, approximately unbiased probabilities with multiscale bootstrap resampling calculated by the pvclust package (Suzuki and Shimodaira 2006).

Next we examined the gene expression profiles of the six RNA mixtures that mimic an actual use in cellular profiling. One typical analysis is to identify genes that are differentially expressed between two biological samples. Here we performed a differential analysis to identify genes expressing higher in HeLa than THP-1 cells. Among 11,924 genes detected by all the technologies, 1701 genes are detected as significantly up-regulated in at least one platform, and 652 of them (∼38%) are detected in common (Fig. 2A). In comparison, in the case of HeliScopeCAGE and microarrays (Kanamori-Katayama et al. 2011), we demonstrated that the difference between the technologies comes largely from inaccurate gene models, that is, TSSs and isoforms of gene models are not necessarily true in all cell types. This explains the difference between CAGE and RNA-seq, in addition to the difference between CAGE and microarray. We will discuss this point again in the section below.

Next we examined the quantification of the panel of RNA mixture. As a comparison with a conventional technology, qRT-PCR, we selected three genes (*CPS1*, *TM4SF1*, *TIMP4*) enriched in HeLa cells, and found that they are consistently quantified with CAGE and RNA-seq as well as conventional technologies such as microarrays and qRT-PCR (Supplemental Fig. S2). Further, we tested whether they can be used to measure similarity or distances between transcriptome states. We performed unsupervised clustering of the six RNA profiles based on Spearman’s correlation coefficient, which reflects similarity relationships between individual profiles (Fig. 2B). For example, the THP-1 RNA 100% pool is the closest to a 1% mixture of HeLa cells in any platform. The hierarchical relationships are identical across the two CAGE platforms, and the hierarchical structure based on the HeliScopeCAGE profiles is robust in computational resampling (Fig. 2B). Notably, the hierarchical structure of the microarray-based clustering is different from the others, where a 5% mixture of HeLa cells is the closest to 10%. This does not reflect the actual mixing ratio, since the 5% mixture has just a 4% difference from the 1% mixture. This result demonstrates a difficulty in measuring the accurate distance between the transcriptome profiles based on a microarray. Although it is not possible to assess this point in RNA-seq from our experimental design, we expect that RNA-seq has a similar performance to HeliScopeCAGE based on its consistency with HeliScopeCAGE, as shown below.

### CAGE and RNA-seq profiles

**Figure 3.**
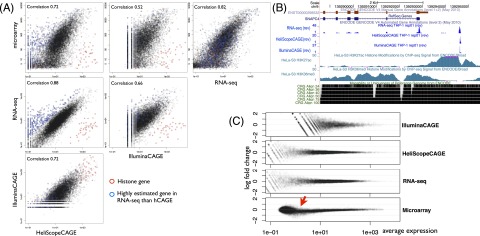
Gene expression quantification by using different platforms. (*A*) Scatter plot of gene expressions by using different platforms, where RPKM (reads per kilobase of exon models per million) is used for RNA-seq. The Spearman’s correlation coefficient is shown for individual comparisons. (*B*) Individual profiles in *SNAPC4* locus. Blue signals indicate reverse-strand signals by the CAGE and RNA-seq platforms. ENCODE histone modification profiles indicating promoter and elongation activities are shown *below*. (*C*) MA plot between the 50% mixture experimental profile and the computationally synthesized one from the THP-1 and HeLa profiles. The 50% profile is based on the average of triplicates, while the computational one is based on combining six profiles (triplicates of THP-1 and HeLa).

Gene expressions obtained from different technologies theoretically should agree if they measure the same materials. We asked if the profiled gene expressions are comparable with each other in HeLa cells (Fig. 3A). As expected, the lowly expressed genes are poorly quantified with our IlluminaCAGE profile. This can be explained by the shallowness of the sequencing depth, where its minimum unit of profiling becomes much lower by the adoption of barcodes. The microarray shows saturation for highly expressed genes in comparison with CAGE and RNA-seq due to its detection method based on hybridization to probes. This may explain the difficulties in monitoring sensitive distances between RNA profiles, as shown above.

Interestingly, HeliScopeCAGE and RNA-seq show the best agreement across the technologies, even though they employ distinct sequencers to determine different parts of RNA molecules. We found that a majority of genes quantified at a very similar level in both platforms (Fig. 3A, black dots), and the rest were clear outliers (Fig. 3A, red and blue dots). After manual inspection, it turned out that all of the CAGE enriched outliers (Fig. 3A, red dots) were histone genes that do not have polyA at their 3′ end. This is totally consistent with the scopes of individual protocols, where CAGE uses total RNA selected by cap regardless of polyA structure, while polyA-selected RNA is used for RNA-seq. The RNA-seq enriched outliers (blue dots in Fig. 3A) likely come from inaccurate gene models in most of the cases. For example, transcriptional initiation of *SNAPC4* is far (1k–2k bp) upstream of the RefSeq and GENCODE gene model 5′ ends (Fig. 3B). CAGE finds a peak upstream, which is supported by ChIP-seq for H3K27 acetylation performed by the ENCODE Project (Ram et al. 2011). Such inaccuracies of gene models are also observed in complex loci, where multiple genes are annotated closely on the same strand. *AHRR* is one of the RNA-seq enriched genes (Fig. 3A, blue dots), and its promoter does not have substantial peaks of CAGE and H3K27 acetylation signals, while its 3′ end has some signals of RNA-seq (Supplemental Fig. S3). Instead, its upstream gene, *PDCD6*, is obviously transcribed according to the CAGE, RNA-seq, and ChIP-seq signals. Interestingly, an RNA polymerase II elongation mark, H3K36 trimethylation, continues from *PDCD6* to *AHRR* gene bodies, and several peaks of H3K27 acetylation are observed in *AHRR* introns. It is clear that *AHRR* is not transcribed—as documented by the gene model—and we can expect read-through of RNA polymerase II from *PDCD6* to the *AHRR* region, or a novel transcriptional initiation site in the *AHRR* introns. Taken together, the gene model–based inconsistencies between CAGE and RNA-seq imply inaccurate gene models rather than technological incompatibility. They demonstrate the complementarity of these two technologies, and their combination will contribute to accurate monitoring of the complex transcriptome that is indicated by deep characterization of different cellular compartments in the ENCODE Project (Djebali et al. 2012).

Next we asked if gene expression is quantified linearly. We tried to mix the two RNA profiles (THP-1 and HeLa cells) computationally and checked if such a computationally mixed profile is consistent with the experimentally mixed one. Surprisingly, a computational mix of 70% THP-1 and 30% HeLa more closely matched the experimentally observed profile of a 50:50 mix of the two RNAs (seen with all platforms, Supplemental Fig. S4). This could be explained by the different complexity of quantified transcripts (Supplemental Fig. S11) or the different ratios of nonquantified (but contained in the RNA extracts) RNA molecules, such as ribosomal RNA or RNA transcribed from the genomic regions that do not appear in the reference genome sequences, such as genomic rearrangement seen in cancer cell lines such as HeLa. Computational mixing with this ratio is very close to the experimentally mixed profile (Fig. 3C) with all the platforms. Notably, only the microarray profiles are skewed at the lowly expressed genes (Fig. 3C, red arrow), which suggests that a microarray can quantify the transcriptome linearly within a limited range of expression levels (approximately three orders of magnitude), whereas the other sequencing platform shows linear quantification with a full range of expression levels. The result of microarray nonlinearity is consistent with a previous study (Shen-Orr et al. 2010).

### Transcript quantification by CAGE employing the second- and third-generation sequencers

**Figure 4.**
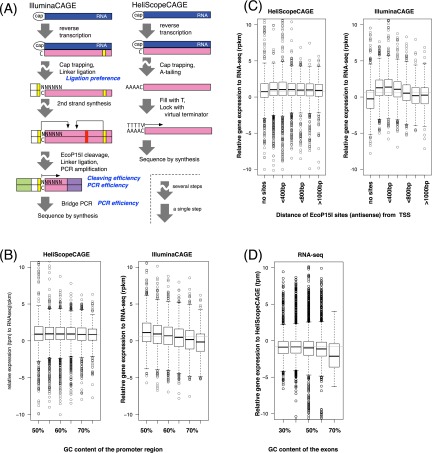
Systematic bias at the gene level. (*A*) Schematic view of the CAGE platforms. Blue box indicates RNA; pink box, DNA; yellow box, EcoP15I sites; red box, internal EcoP15I sites; and green and purple boxes, 5′ and 3′ linkers. Text in blue suggests potential causes of gene quantification bias. (*B*) Relative expression of the CAGE profiles against RNA-seq quantification, according to GC content within 500 bp from RefSeq TSS. (*C*) Relative expression of the CAGE profiles depending on the presence of EcoP15I sites on an antisense strand to the gene orientation. (*D*) Relative expression of the RNA-seq profiles against HeliScopeCAGE quantification, according to GC content of the exons.

One of the unique points in this study is a comparison between the CAGE protocols optimized for second- and third-generation sequencers. Both of them employ the cap-trapping step to select the 5′-end–capped site of long RNAs (Carninci et al. 1996), but they treat the resulting cDNAs differently afterward (Fig. 4A), and their results of sequencing can be different depending on reverse transcriptase activities, which can add nontemplated bases to cDNAs at the cap-site (Chen and Patton 2001). The scatter plot between HeliScopeCAGE and IlluminaCAGE (Fig. 2A) suggests that their gene expression profiles are largely consistent; however, the agreement is not very precise. This can be explained by protocol differences as well as sequencing depth. Here we examine systematic differences that cannot be explained by the sequencing depth only.

First, we asked if GC content could introduce any differences since it is reported that PCR amplification efficiency depends on GC content (Kozarewa et al. 2009) and elevated error rates occur in GC-rich sequences on the Illumina platform (Dohm et al. 2008; Nakamura et al. 2011). Relative gene expression profiles of the two CAGE technologies against RNA-seq demonstrate that the GC contents clearly affected the IlluminaCAGE profiles but not HeliScopeCAGE (Fig. 4B).

Since PCR amplification and Illumina sequencing are also employed in the RNA-seq platform, we asked if RNA-seq quantification is affected in a similar way as IlluminaCAGE. Relative gene expression levels quantified by RNA-seq in comparison with HeliScopeCAGE (Fig. 4D) indicate that RNA-seq results are also biased by GC content. These results demonstrate that sequencing-based analysis relying on PCR amplification steps, used in both IlluminaCAGE and RNA-seq, quantifies gene expression levels reproducibly, but that the quantified levels are biased by GC content. Conversely, a PCR-free protocol, HeliScopeCAGE, successfully overcomes such biases.

Second, we examined whether RNA target molecules that contained EcoP15I recognition sites (Hadi et al. 1979), a type III restriction enzyme used to generate 27 base tags in the Illumina tag protocol, were biased compared with transcripts that lacked these sites. The IlluminaCAGE protocol employs EcoP15I to obtain 5′-end cDNA molecules with a fixed length, where EcoP15I recognizes the sequence within the 5′ linker and cleaves 25–27 bp downstream from the recognition site (Fig. 4A; Takahashi et al. 2012). Native EcoP15I sites harboring in the target RNA molecules could potentially affect the cleavage efficiency; however, this possibility has not yet been examined. We examined relative expression levels against RNA-seq depending on the existence of a EcoP15I site, and found that internal recognition sites indeed disrupt quantification of RNA abundance (Fig. 4C). Quantified gene expression levels are overestimated when internal recognition sites are located in the reverse orientation and within 400 bp from the TSS, which can be interpreted as the internal restriction site increases the chance of observation in sequencing results by providing an additional opportunity of cleavage.

### TSS activities at a single-base-pair resolution by the CAGE platforms

**Figure 5.**
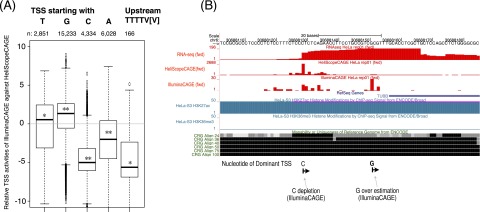
Systematic bias at a single-base-pair resolution. (*A*) Relative TSS activities of IlluminaCAGE against HeliScopeCAGE. (*B*) The CAGE and RNA-seq profiles on the *TUBB* promoter.

We found that the CAGE platforms are less consistent at the TSS level than at the gene level (Supplemental Fig. S5), while they are correlated with each other at the gene level and their technical reproducibility is quite high even at the TSS level (as shown in Fig. 1C). We asked if there exist any systematic differences that depend on the sequence around the transcriptional initiation site. Because of the template-free activity of the reverse transcriptase used to prepare the cDNA, an additional G nucleotide is often attached to the 5′ side (Supplemental Fig. S6), and a previous study estimated the probability as ∼87% (Carninci et al. 2006). Such an additional G cannot be distinguished as an artifact or not when G is encoded at 1 bp upstream of the TSS in the genome, unless performing an active correction (Carninci et al. 2006). This base addition is rarely observed in sequences produced by HeliScopeCAGE (Fig. 4A; Supplemental Fig. S6) due to “fill and lock” treatment of the DNA template immediately before sequencing (Harris et al. 2008). On the other hand, in case that base addition does not happen during reverse transcription, identified TSSs can be shifted one or more bases downstream, depending on the starting bases, in HeliScopeCAGE (Supplemental Fig. S6). The examination of TSS profiles depending on the starting bases suggests that both can occur (Fig. 5A). G-starting TSSs are overestimated in IlluminaCAGE, and downstream shifting after a T stretch happens in HeliScopeCAGE. The extent of the difference is remarkable in T-stretch shifting, but the effect is quite limited to a small fraction of the TSSs (0.6%, 166 of 28,446 TSSs). Unexpectedly, we found an underestimation of C-starting TSS activities, which is remarkable in comparison with A- and T-starting ones. This cannot be explained by overestimation of G-starting TSSs, and one potential interpretation would be different efficiency of 5′ linker ligation. The IlluminaCAGE protocol uses two types of linker sequences, 20% of random six nucleotides and 80% of G plus random five nucleotides (Supplemental Fig. S7; Takahashi et al. 2012), to supply enough amounts of 5′ linkers in the ligation step. One could hypothesize that domination of G-starting linkers decreased the probability to capture C-starting TSSs, while we cannot reject other possibilities without further experiments. These differences can be found at individual loci. At the TSS region of tubulin, beta class I (*TUBB*) (Fig. 5B), HeliScopeCAGE identified one striking TSS starting with C, while IlluminaCAGE identified a downstream TSS starting with G as dominant. RNA-seq supports the HeliScopeCAGE profile, since it suggests the presence of abundant RNA up to the upstream C-starting TSS. In the case of protein phosphatase 1, regulatory subunit 15B (*PPP1R15B*), we can see a case of T-stretch shifting in HeliScopeCAGE as well as C depletion and G overestimation in IlluminaCAGE (Supplemental Fig. S8). While we found systematic differences at a single-pair resolution, averaging over three bases (upstream and downstream one base) improves the correlation significantly (from −0.2 to 0.2). This is consistent with the screenshots (Fig. 5B; Supplemental Fig. S8), in which identified TSSs are largely consistent with each other, while TSS shapes are slightly different.

Last, we examined whether the two CAGE platforms identified the same transcription initiation events. Since the depth of sequencing is totally different, here we asked if all IlluminaCAGE TSSs have been found by HeliScopeCAGE or not. Of about 90,000 TSSs identified with more than 10 counts at least in a single profile, 0.5% (467) are found only by IlluminaCAGE. Just 0.04% (35) was not neighbored by HeliScopeCAGE’s TSS with three or more counts (Supplemental Fig. S9). Manual inspection of the 35 TSSs revealed two major classes (Supplemental Table S2): G-starting ones, which are likely to be favored by the IlluminaCAGE, and T-rich 5′-end sequences, which can be misidentified as artifacts by filterSMS (see Methods below). Taken together, the CAGE platforms identified almost the same transcription initiations overall, while favored TSSs are different depending on the platforms.

## Conclusion

We systematically investigated several sequencing-based transcriptome profiling platforms: IlluminaCAGE, HeliScopeCAGE, and RNA-seq. The results demonstrate their performance. Their reproducibility is quite high even at the gene level or individual TSS levels. Quantified RNA levels are comparable across the platforms; however, we found that GC content affects IlluminaCAGE and RNA-seq measurements, most likely due to employment of PCR amplification in their protocols. We also found that naturally encoded EcoP15I sites at the 5′ end of RNA molecules influenced IlluminaCAGE measurements, presumably due to competition between the EcoP15I sites in the cDNA affecting tag cleavage efficiencies. The HeliScopeCAGE protocol, which relies on single-molecule sequencing technology, quantifies gene expression levels without PCR-amplification biases.

TSS activities at the ultimate single-base-pair resolution, which are quantifiable only on the CAGE platforms, are reasonably reproducible within replicates of a single platform; however, they are less consistent across different platforms than are gene expression levels. This can be explained by the efficiency of linker ligation, template-free G addition by reverse transcriptase, and the “fill and lock” steps of single-molecule sequencing. While the fraction of affected TSSs in HeliScopeCAGE is very minor (<1%), the result indicates that it is still challenging to quantify TSS activities at a single-base-pair resolution without any systematic bias. Nevertheless, it should be noted that the identified TSSs and their activities are largely consistent across the platforms, and even the slightly biased levels of TSS activities by IlluminaCAGE were effectively used in quantitative modeling of transcription based on epigenetic marks (The ENCODE Project Consortium 2012).

Last, the in-depth inspection in this study demonstrates that the combination of CAGE and RNA-seq enables us to approach unknown variation in transcript structures. Besides the technical performance of these technologies, their complementary application will be crucial for revealing and refining the complexity and estimating the expression levels of individual genes.

## Methods

### Cell culture and RNA preparation

THP-1 cells were cultured in RPMI1640 (Invitrogen) supplemented with 10% FBS, penicillin/streptomycin (Invitrogen), 10 mM HEPES (Invitrogen), 1 mM sodium pyruvate, and 50 μM 2-mercaptoethanol. HeLa cells were cultured in Eagle’s MEM (Invitrogen) supplemented with 10% FBS, 1% NEAA (Invitrogen), and penicillin/streptomycin. Total-cell lysates were harvested in TRIzol reagent (Invitrogen); total RNA was purified from TRIzol lysates according to the manufacturer’s instructions; and we used RNase-free glycogen (Invitrogen) as a carrier in the aqueous phase prior to precipitating the RNA with isopropyl alcohol. The RNA extracts were checked by an Agilent 2100 Bioanalyzer, which confirmed their qualities as RIN scores 9.6 and 9.9 for the THP-1 and HeLa RNA extracts. The prepared total RNAs were mixed with the ratio described above. The same RNA extracts previously described (Kanamori-Katayama et al. 2011) were used.

### IlluminaCAGE

We followed the CAGE protocol previously described (Takahashi et al. 2012), and sequenced with Illumina GA IIx, where we used linkers including specific barcodes (the samples and barcodes used here are described in Supplemental Table S1). After base calling, we grouped the reads according to the barcode sequences identifying the RNA source and trimmed the barcode sequence. We removed artifactual sequences originated from adapter linkers with tagdust (Lassmann et al. 2009) and identified the reads matching to ribosomal DNA repeat sequences (U13369) within two mismatches. Only the remaining reads were used for alignment with the human genome assembly (GRCh37), where we employed BWA (Li and Durbin 2009) for the alignment and selected only the alignment with a mapping quality score ≥20. When the alignments harbor mismatches at the 5′ end of the CAGE tags, the mismatched bases are trimmed to identify the starting position of the alignments. CAGE read alignments starting close to known TSSs represent our targeted signals; therefore, we examined the distance distribution of CAGE reads from known TSSs, which we defined as the 5′ end of RefSeq transcripts (Pruitt et al. 2012), to determine the CAGE reads for gene expression analysis. The result (Supplemental Fig. S10) indicates that the signal ratio for the CAGE protocols (IlluminaCAGE as well as HeliScopeCAGE) reaches saturation after 400–500 bp; therefore, we took 500 bp as the threshold for gene expression analysis. That is, we quantified abundance of genes based on the reads aligned within the upstream/downstream 500-bp region from the 5′ end of the RefSeq transcripts. In comparison with microarray analysis, we quantified gene expression by the accumulation of all the signals (read counts or TPMs) corresponding to the gene.

### HeliScopeCAGE

We followed the CAGE protocol previously described (Kanamori-Katayama et al. 2011), and sequenced with HeliScope. As for the THP-1 and HeLa profiles (100%:0% and 0%:100%), we obtained the sequence data previously described (deposited in DRA as DRA000368) (Kanamori-Katayama et al. 2011). Additionally, we took the remaining profiles for this study. Artifactual sequences were filtered out with filterSMS, a tool included in Helicos HeliSphere software, accepting only 20- to 70-nt read lengths, and the remaining reads were aligned with the human genome assembly (GRCh37) by using an in-house alignment program called Delve as previously described (Itoh et al. 2012). It employs a paired hidden Markov model to iteratively map reads to the genome and estimate position-dependent error probabilities, and individual reads are placed in a single position on the genome where the alignment has the highest probability to be true according to the pHMM model. We selected only the alignment with a mapping quality score ≥20 and percentage identity ≥85%. We performed the following analyses—quantification of transcript abundance and gene expression—in the same way as IlluminaCAGE. Overlaps of genes detected by HeliScopeCAGE, RNA-seq, and microarray are shown in Supplemental Figure S12.

### RNA-seq

We constructed sequencing libraries starting from 500 ng of total RNA. We isolated poly(A)^+^ RNA using Dynabeads Oligo(dT)_25_ (Invitrogen) according to the manufacturer’s protocol. This isolation step was repeated two times. Poly(A)^+^ RNA was fragmented by heating for 3.5 min at 70°C in a 0.5× fragmentation buffer (Ambion). Fragment RNA was purified with the RNeasy MinElute kit (Qiagen) following the instructions of the manufacturer except 675 μL of 100% ethanol was used in step 2. Purified RNA was dephosphorylated by adding 2 μL of 10× phosphatase buffer, 5 units of Antarctic phosphatase (NEB), and 40 units of RNaseOUT (Invitrogen) and incubating for 30 min at 37°C followed by 5 min at 65°C. After incubation, the sample was set on ice and 5 μL of 10× PNK buffer, 20 units of T4 polynucleotide kinase (NEB), 5 μL of 10 mM ATP (Epicentre), 40 units of RNaseOUT, and 17 μL of water were added, and incubated at 37°C for 60 min. Phosphorated RNA was purified with the RNeasy MinElute kit (Qiagen) as described before. Purified RNA was concentrated to 6 μL by a miVac DNA concentrator (Genevac). A mixture of 2 μM preadenylated 3′ DNA adaptor and 1 μL concentrated RNA was incubated for 2 min at 70°C and immediately kept on ice for 2 min. One microliter of 10× T4 RNA ligase 2 truncated buffer, 0.8 μL of 100 mM MgCl_2_, 20 units of RNaseOUT, and 200 units of RNA ligase 2 truncated (NEB) were added to make a 10 μL reaction. The reaction was incubated for 60 min at 20°C. One microliter of heat-denatured 5 μM 5′ RNA adapter was ligated with 3′ adapter ligation products with 20 U of T4 RNA ligase 1 (NEB) and 1 μL of 10 mM ATP (NEB) for 60 min at 20°C. We mixed 4 μL of adaptor-ligated RNA with 1 μL of 20 μM RT primer, followed by incubation for 2 min at 70°C and being immediately kept on ice. We synthesized single-stranded cDNA with this RNA primer mix by adding 2 μL 5×PrimeScript buffer, 1 μL of 10 mM dNTP, 20 units of RNaseOUT, and 200 units of PrimeScript reverse transcriptase (Takara) and incubating for 30 min at 44°C. The whole cDNA product is amplified by PCR with 10 μL of 5× HF buffer, 1.25 μL of 10 mM of each dNTP mix, 2 μL of 10 μM FWD primer, 2 μL of REV primer, and 1 unit of Phusion high-fidelity DNA polymerase (NEB). PCR is carried out in a total of 50 μL. After incubation for 30 sec at 98°C, 12 PCR cycles were performed for 10 sec at 98°C, 30 sec at 60°C, and 15 sec at 72°C. Finally, the sample is incubated for 5 min at 72°C and kept at 4°C. We removed PCR primers using 1.2 volumes of AMPure XP beads (Beckman). This step was repeated two times. We sequenced libraries with the Illumina Genome Analyzer II (35-base single read) using the following custom sequencing primers:

pre-adenylated 3′ DNA adaptor, App/ATCTCGTATGCCGTCTTCTGCTTG/3′ idT
5′ RNA adapter, guucagaguucuacaguccgacgaucgaaa
RT primer/REV primer, CAAGCAGAAGACGGCATACGA
FWD primer, AATGATACGGCGACCACCGACAGGTTCAGAGTTCTACAGTCCGA
sequencing primer, CGACAGGTTCAGAGTTCTACAGTCCGACGATCGAAA

After base calling, we removed artifactual sequences originated from adopter linkers with TagDust (Lassmann et al. 2009), and identified the reads matching to ribosomal DNA repeat sequences (U13369) within two mismatches. Only the remaining reads are used for alignment with the human genome assembly (GRCh37), where we employed BWA (Li and Durbin 2009) for the alignment and selected only the alignment with a quality score ≥20. Quantification of transcript abundance and gene expression levels is based on the reads aligned within exons of RefSeq transcripts (Pruitt et al. 2012).

### Microarray

Five hundred nanograms of total RNA was amplified using the Illumina TotalPrep RNA amplification kit (Ambion), according to manufacturer’s instructions. cRNA was hybridized to Sentrix Human-6 Expression BeadChips v3 (Illumina), according to standard Illumina protocols. Chips scans were processed using the Illumina BeadScan and BeadStudio software packages, and summarized data were generated in BeadStudio (version 3.4). We used the lumi (Du et al. 2008) and Limma (Smyth 2004) packages for normalization and differential analysis of detected intensities by individual probes. In the comparison of gene expression levels with other platforms, we averaged all the probe signals to the corresponding gene. As for the THP-1 and HeLa profiles (100%:0% and 0% and 100%), we used the same data obtained by Kanamori-Katayama et al. (2011) (deposited in GEO as GSE28148).

### Quantitative reverse transcription-polymerase chain reaction (qRT-PCR)

Reverse transcription of the total RNA was achieved with PrimeScript reverse transcriptase (Takara) and random hexamer in accordance with the manufacturer’s protocol. The PCR primer sequences from this analysis are given in Supplemental Table S3. PCR amplification was performed on the ABI PRISM 7900HT system (Applied Biosystems). For amplification, SYBR Premix Ex *Taq* II (Takara) was used as instructed in the manufacturer’s manual. The PCR conditions were an initial step of 10 sec at 95°C, followed by 40 cycles of 3 sec at 95°C and 20 sec at 62.5°C.

## Data access

The sequencing data obtained for this study have been submitted to the DDBJ Read Archive (http://trace.ddbj.nig.ac.jp/dra/index_e.shtml) under accession number DRA001100. This work is part of the FANTOM5 project. Data downloads, genomic tools, and copublished manuscripts are summarized at http://fantom.gsc.riken.jp/5/. Supplemental data are accessible at http://fantom.gsc.riken.jp/5/suppl/Kawaji_et_al_2013 as a part of the FANTOM web resource.
